# Association of Blast‐induced Hearing Loss with Progression of Alzheimer’s Disease Pathology

**DOI:** 10.1002/alz.089629

**Published:** 2025-01-03

**Authors:** Nadine A Kerr, Rachele Sangaletti, Winston Walters, Suhrud M Rajguru

**Affiliations:** ^1^ The Miami Project to Cure Paralysis, Miami, FL USA; ^2^ University of Miami, Miami, FL USA; ^3^ Bruce W. Carter Department of Veterans Affairs Medical Center, Miami, FL USA

## Abstract

**Background:**

Exposures to hazardous noise causes irreversible injury to the structures of the inner ear, leading to changes in hearing and balance function with strong links to age‐related cognitive impairment. While the role of noise‐induced hearing loss in long‐term health consequences, such as progression or development of Alzheimer’s Disease (AD) has been suggested, the underlying mechanisms and behavioral and cognitive outcomes or therapeutic solutions to mitigate these changes remain understudied. This study aimed to characterize the association between blast exposure, hearing loss, and the progression of AD pathology, and determine the underlying mechanisms.

**Method:**

We employed a well‐accepted preclinical mouse model (3xTg) with a predisposition to AD pathology. Wild type (WT) and 3xTg (AD) mice were exposed to blasts in an ecologically valid oxyacetylene gas tube at 4 months of age. We compared functional outcomes (auditory brainstem responses and vestibular evoked myogenic potentials), cognitive deficits (novel object recognition, water maze), affective deficits (open field) and anxiety between WT and AD sham and blast‐exposed mice over 3 months. We also carried out immunohistochemical and Western Blot analysis for amyloid beta (Ab) amyloid fibrils, Tau, and Gasdermin‐D of the peripheral auditory system and multiple brain regions including auditory cortex and brainstem.

**Result:**

Auditory and vestibular analyses revealed a combination of temporary and permanent functional loss post‐blast combined with hair cell loss and synaptopathy. We assessed changes in cognitive function and anxiety after the induction of hearing loss in WT and AD mice. The AD, WT‐blast and AD‐blast animals had challenges with the novel object recognition. AD mice post‐blast exhibited significant increases in fecal boli present when compared to WT mice. In open field, there was a decrease in overall mean speed and total distance traveled in AD mice. Finally, there was significant elevation of Ab, Tau and Gasdermin in the cochlea as well as brainstem and cortex suggesting that pyroptosis related mechanisms play a significant role in the pathology of AD.

**Conclusion:**

These results suggest that strong association between early hearing loss and progression of AD pathology. Our novel results may inform potential biomarkers and therapeutic strategies.

